# Complete Genome Sequence of *Streptococcus pneumoniae* Strain A026, a Clinical Multidrug-Resistant Isolate Carrying Tn*2010*

**DOI:** 10.1128/genomeA.01034-13

**Published:** 2013-12-12

**Authors:** Zhihai Sui, Wenqing Zhou, Kaihu Yao, Li Liu, Gang Zhang, Yonghong Yang, Jie Feng

**Affiliations:** State Key Laboratory of Microbial Resources, Institute of Microbiology, Chinese Academy of Sciences, Beijing, Chinaa; Beijing Pediatric Research Institute, Beijing Children’s Hospital, Capital Medical University, Beijing, Chinab

## Abstract

*Streptococcus pneumoniae* is a primary cause of bacterial infection in humans. Here, we present the complete genome sequence of *S. pneumoniae* strain A026, which is a multidrug-resistant strain isolated from cerebrospinal fluid.

## GENOME ANNOUNCEMENT

*Streptococcus pneumoniae*, also known as pneumococcus, commonly colonizes the human upper respiratory tract. It is also a leading cause of bacterial infection, including serious invasive disease in young children and elderly people. The World Health Organization (WHO) estimates that 1.6 million people die of pneumococcal disease annually, with 0.7 to 1 million deaths in children less than 5 years of age, mostly in developing countries (1). To date, more than 92 serotypes of *S. pneumoniae* have been recognized (2). Globally, serotype 19F was one of the most common serotypes before the universal administration of pneumococcal conjugate vaccine (PCV) (3, 4). The PCV7 immunization became available to the private sector in China in September 2008. The immunization coverage rate is limited because of the high cost (860 renminbi [RMB] per dose). This serotype, therefore, is still frequent and associated with antibiotic resistance in China (5, 6). In our previous study, the majority of serotype 19F isolates were identified as being from sequence type 271 (ST271) (7). ST271 is a single-locus variant (SLV) of the globally spread Taiwan^19F^-14 clone (http://www.sph.emory.edu/PMEN/index.html).

*S. pneumoniae* A026 (ST271, serotype 19F) is a multidrug-resistant strain isolated from cerebrospinal fluid in pediatric clinical work. The genome sequencing of A026 was performed using the Illumina HiSeq 2000 system. A total of 11.78 million high-quality 101-bp paired-end reads were produced, yielding appreciable coverage of approximately 504×. Assembly was performed using the Velvet software (8) and resulted in 14 contigs. The contig N_50_ is 367,731 bp, and the largest contig assembled was 555,527 bp. The assembled contigs were ordered and oriented into scaffolds by alignment with the *S. pneumoniae* Taiwan^19F^-14 genome sequence using the Mauve software (9). Gaps between the scaffolds were confirmed and closed by PCR amplification and primer walking. The A026 genome contains 2,091,879 nucleotides, with a 39.76% G+C content. A total of 2,082 predicted protein-coding sequences (CDSs) were detected by the NCBI Prokaryotic Genomes Automatic Annotation Pipeline. In addition to the CDSs, 58 tRNA and 12 rRNA genes were identified.

A026 is resistant to erythromycin, tetracycline, penicillin, trimethoprim, and sulfonamides but is susceptible to levofloxacin and vancomycin. The A026 genome contains the transposon Tn*2010*, which harbors three resistance determinants [*erm*(B), *mef*(E), and *tet*(M)]; this might explain its erythromycin and tetracycline resistance (10). In a sequence analysis of A026, many mutations that can confer antibiotic resistance were identified. The amino acid change Ile-100-Leu of the dihydrofolate reductase (DHFR) in A026 might result in trimethoprim resistance (11), while the duplication of amino acids 58-Arg and 59-Pro of the dihydropteroate synthase (DHPS) might cause sulfonamide resistance (12). In addition, mutations conferring β-lactam resistance were found in *pbp2a*, *pbp2b*, and *pbp2x*, and these mutations are identical to those in β-lactam-resistant clinical *S. pneumoniae* isolates in previous studies (13).

### Nucleotide sequence accession number.

The complete genome sequence of *Streptococcus pneumoniae* strain A026 has been deposited in GenBank under the accession no. CP006844.
